# Knockdown of annexin A2 enhances the host cell apoptosis induced by *Eimeria tenella*

**DOI:** 10.3389/fvets.2025.1595384

**Published:** 2025-07-24

**Authors:** Jixia Wang, Mingxiao Wang, Yuting Wang, Mengbo Xu, Yang Liu, Mingxue Zheng, Rui Zhao, Rui Bai, Yanping Zhao, Li Zhang, Xiaoling Lv, Yu Yang, Wenchao Guan, Xiaozhen Cui

**Affiliations:** ^1^College of Veterinary Medicine, Shanxi Agricultural University, Jinzhong, China; ^2^College of Animal Science, Shanxi Agricultural University, Jinzhong, China

**Keywords:** *Eimeria tenella*, cell apoptosis, RNA interference, primary cell culture, annexin A2

## Abstract

Annexin A2 (ANXA2) is a multifunctional protein involved in host-pathogen interactions during viral and parasitic infections. To investigate the role of ANXA2 in host cell apoptosis induced by *Eimeria tenella*, RNA interference (RNAi) was employed to specifically downregulate ANXA2 expression. Primary cultures of chicken embryonic cecal epithelial cells were established and subjected to *E. tenella* sporozoite infection. A comprehensive analytical approach integrating hematoxylin-eosin staining, Hoechst-Annexin V-PI triple-staining, and caspase-3 activity quantification was used. Western-blot and RT-qPCR were performed to assess transcriptional and translational changes in key apoptosis-related factors, including B-cell lymphoma (Bcl-2) and Bcl-2-associated X protein (Bax). Additionally, the dynamic expression of ANXA2 was analyzed to clarify its function in the parasite-host interaction. The results showed that the ANXA2 expression in the *E. tenella* group increased at 4 h after inoculation but decreased at 24 to 96 h compared to the control group (*P* < 0.01). Following ANXA2 knockdown, the cell apoptosis rate, caspase-3 activity, and Bax expression levels were significantly increased (*P* < 0.01), whereas the infection rate and Bcl-2 expression levels were significantly decreased (*P* < 0.01) compared to the group infected with *E. tenella* alone. In conclusion, ANXA2 serves as a critical regulator of host cell responses during *E. tenella* infection. RNAi-mediated suppression of ANXA2 expression significantly enhances apoptosis induced by *E. tenella*. This study establishes a foundation for further exploration of therapeutic targets to reduce host tissue damage, indicating that targeting ANXA2 may be a viable approach for controlling coccidiosis.

## 1 Introduction

Coccidiosis in chickens, caused by the intracellular parasite *Eimeria*, is a significant disease that targets the intestinal tract and results in substantial economic losses to the poultry industry (1–4). Among the seven common species of *Eimeria, Eimeria tenella* (*E. tenella*) is particularly pathogenic and prevalent (5), primarily affecting the cecal mucosa and adjacent intestines of chickens, leading to inflammation, cell death, and shedding of mucosal epithelial cells (6–9). Moreover, the infection process exacerbates apoptosis of cecal epithelial cells in affected chickens (7, 10). Currently, coccidiosis is primarily controlled through the use of anticoccidial drugs and vaccines. However, issues such as drug resistance, drug residues, and vaccine side effects have become increasingly prominent (11–15). Therefore, it is imperative to identify new targets for the prevention and control of coccidiosis. Recent studies have begun to elucidate the mechanisms by which coccidial proteins regulate apoptosis in host cells. For example, the epidermal growth factor-like (EGF-like) domain in *E. tenella* microneme protein 4 is significantly associated with triggering the epidermal growth factor receptor (EGFR), cell proliferation, and apoptosis (16). *E. tenella* rhoptry protein 38 inhibits the initiation of the p38 mitogen-activated protein kinase (MAPK) pathway in host cells, thereby suppressing cell apoptosis (17). Additionally, *E. tenella* uses apical membrane antigen 1 to inhibit both death receptor-mediated and mitochondrial apoptotic pathways, promoting parasite persistence (18). *E. tenella* can promote its own development and proliferation by modulating host cell apoptosis (19–21). However, the specific mechanisms through which *E. tenella* infection induces apoptosis of host cells remain incompletely understood.

Annexin A2 (ANXA2) is a multifunctional protein involved in various biological processes, including vesicular transport, signal transduction, cell migration, transcription, mRNA transport, and apoptosis (22–25). It has been implicated in the infection mechanisms of several pathogens and is considered a key host protein for successful pathogen invasion (26–30). For instance, the porcine reproductive and respiratory syndrome virus (PRRSV) infection results in increased ANXA2 levels in porcine alveolar macrophages. ANXA2 expression in Marc-145 cells is notably diminished through application of small interfering RNA (siRNA), which significantly hinders PRRSV replication (31). ANXA2 promotes the replication and airborne transmission of H9N2 avian influenza virus (AIV) by mediating the conversion of plasminogen into plasmin (32). Metacyclic trypomastigotes of the G strain of *Trypanosoma cruzi*, which express the mucin molecules Gp35/50 on their surface, bind to ANXA2 on host cells, activating protein tyrosine kinases and actin rearrangement to facilitate parasite entry (33). Galectin-1 from the *Angiostrongylus cantonensis* can induce macrophage apoptosis through its interaction with ANXA2, subsequently activating JNK in the downstream signaling pathway associated with apoptosis (34). Furthermore, ANXA2 has been shown to interact with *Eimeria tenella* serine protease inhibitor 1 (*Et*Serpin1) and function as a membrane receptor, partially inhibiting parasite invasion into host cells. These findings highlight the pivotal role of ANXA2 in *E. tenella* infection. However, the specific contribution of ANXA2 to *E. tenella*-induced host cell apoptosis remains unclear and requires deeper exploration.

This study established an *in vitro* primary culture model of chicken embryonic cecal epithelial cells, employed RNA interference (RNAi) to knockdown ANXA2, and assessed apoptosis-related parameters following infection with *E. tenella* sporozoites. The aim was to elucidate the effects of ANXA2 silencing on host cell apoptosis induced by *E. tenella*.

## 2 Materials and methods

### 2.1 Animals and parasites

Fourteen-day-old specific-pathogen-free (SPF) chicken embryos were obtained from the Beijing Meri Avigon Laboratory Animal Technology Co., Ltd. (Beijing, China). The *E. tenella* Shanxi strain was provided by the Veterinary Pathology Laboratory, Shanxi Agricultural University, and maintained in 2.5% potassium dichromate solution.

### 2.2 Isolation and culture of chicken embryo cecal epithelial cells

Cecal epithelial cells were cultured following the previously established technique of primary cell culture (35). In summary, the cecum was gently extracted from chicken embryos and subsequently washed in PBS containing a penicillin-streptomycin mixture (Meilunbio, China; Cat. MA0110) at 41°C to remove the blood and other contaminants. The cecum was cut into tissue blocks of ~1 mm^3^ using sterile surgical scissors to increase the surface area for cell release. The tissue blocks were then transferred to thermolysin (Sigma, USA; Cat. T7902) and incubated for 40–60 min, rotating at a speed of 80 r/min to facilitate cell separation. Utilizing the differential adhesion rates of various cell types, the cell precipitates were resuspended in cell culture medium supplemented with 10% fetal bovine serum (FBS) (Cellmax, USA; Cat. SA301.02.V), seeded into disposable cell culture flasks, and incubated in an 8% CO_2_ environment at 41°C for 40 min to isolate different cells. Floating cells in the culture plates were gathered and subjected to centrifugation to discard the medium. The cell growth medium consisted of DMEM (Boster, China; Cat. PYG0072) supplemented with L-glutamine, sodium heparin, sodium pyruvate, penicillin-streptomycin mixture, hyperinsulin, EGF, and FBS. Cells were cultured in 6-well plates and round coverslips were placed in selected wells. The cell culture plates were maintained at 41°C in an incubator with 8% CO_2_.

### 2.3 RNA interference

The siRNA used in this study was developed and produced by Sangon Biotech (Shanghai, China). The siRNA targeting *ANXA2* was designed based on the chicken *ANXA2* mRNA sequence (GenBank NM_205351.2). Follow the protocol provided with the Lipofectamine RNAiMAX Reagent kit (Invitrogen, USA; Cat. 13778150) for transfection. The cell adhesion rate was observed, and transfection reagent preparation was initiated when the cell adhesion reached 80%. Both siRNA *ANXA2* and negative control (NC) siRNA were diluted to a final concentration of 60 nM. The siRNA sequence is shown in Table 1.

**Table 1:** siRNA sequences.

**Name**	**Sequence**	
siRNA ANXA2	Sense	GAUGCUGGUGUCAAGAGAATT
	Antisense	UUCUCUUGACACCAGCAUCTT
NC siRNA	Sense	UUCUCCGAACGUGUCACGUTT
	Antisense	ACGUGACACGUUCGGAGAATT

### 2.4 Preparation of *E. tenella* sporozoites

Coccidial oocyst amplification was achieved by oral inoculation of sporulated oocysts into 14-day-old SPF chicks and collecting feces on the 8th day after infection. The purification of *E. tenella* sporozoites followed previously reported methods with improvements (36). Sporocysts were released from oocysts using a vortex shaker at a ratio of glass beads to sporulated oocysts of 1:1. Sporozoites were released by digesting the walls of the sporocysts with bile salt-trypsin solution preheated at 37°C. Pure sporozoites were obtained by filtration using a G3 sand core funnel. After 48 h of transfection with siRNA *ANXA2*, the cells were inoculated in 6-well plates at a concentration of 4.0 × 10^5^ sporozoites/well.

### 2.5 Experimental protocol *in vitro*

Chicken embryo cecal epithelial cells, cultured in 6-well plates (including round coverslips), were randomly assigned to several groups: *E. tenella* group, siRNA *ANXA2* group, siRNA *ANXA2* + *E. tenella* group, NC siRNA + *E. tenella* group, and control group (untreated cells), when cell adherence rate reached 80%. Each group contained three biological replicates. Cells were collected at 4 h (sporozoite invasion stage), 24 h (trophozoite stage), and 96 h (schizont stage) after inoculation with sporozoites to detect relevant indicators.

### 2.6 Hematoxylin and eosin (H&E) staining

At the designated time points following infection with *E. tenella* sporozoites, the round coverslips were removed and subsequently stained with H&E according to a previously reported approach (17). Infections caused by *E. tenella* were assessed via light microscopy in 200 selected cells. The sporozoite infection rate (%) was calculated as: (number of infected cells/200) × 100.

### 2.7 Trichromatic fluorescence

Cells from each group were digested with 0.25% trypsin (37°C, 5 min), and digestion was terminated by adding complete medium. After centrifugation, cells were washed twice with pre-cooled PBS and resuspended in binding buffer. Apoptosis was assessed using Hoechst 33342 (Beyotime, China; Cat. C1025) and an Annexin V-FITC/PI double-staining kit (BD Biosciences, San Diego, CA; Cat. 556547). Cells were first incubated with Hoechst 33342 (100 μL) for 20 min at 37°C in the dark, followed by Annexin V-FITC/PI according to the manufacturer's protocol. After centrifugation, cells were resuspended in binding buffer and observed under a fluorescence microscope (Olympus, Hatagaya, Japan). Nuclear morphology and fluorescence signals were categorized as follows: all cell nuclei were stained blue by Hoechst 33342. Cells bound to Annexin V-FITC exhibited green fluorescence on the plasma membrane, while loss of membrane integrity was indicated by red nuclear staining (PI+). Cells were defined as follows: normal cells (Annexin V-/PI-); early apoptotic cells (Annexin V+/PI-); and late apoptotic and necrotic cells (Annexin V+/PI+). For each time point, five parallel samples were analyzed, with at least 200 cells counted per sample to calculate the apoptotic cell percentage.

### 2.8 RT-qPCR

Total RNA was extracted from cells using the guanidine isothiocyanate-phenol-chloroform method. Trizol (Takara Bio, Japan; Cat. 9018/9019) was used to lyse the cells, after which chloroform was added to extract nucleic acids. RNA was precipitated with isopropanol, washed twice with 75% ethanol, air-dried, and finally dissolved in RNase-free water. cDNA synthesis was performed using a PrimeScript RT reagent kit (Takara Bio, Japan; Cat. RR092S). Gene expression was quantified by RT-qPCR with TB Green Premix Ex Taq (Takara Bio, Japan; Cat. RR820A), and relative expression levels were normalized using the 2^−Δ*ΔCT*^ method. The primer details are listed in Table 2.

**Table 2:** Sequences of the primers used for the RT-qPCR assay.

**Gene name**	**Primer sequences**	**Product length (bp)**	**GenBank accession No**.
β-actin-F	5′-CACCACAGCCGAGAGAGAAAT-3′	135	L08165.1
β-actin-R	5′-TGACCATCAGGGAGTTCATAGC-3′		
Bcl-2-F	5′-AGGACAACGGAGGATGGGATG-3′	109	NM_205339
Bcl-2-R	5′-ACCAGAACCAGGCTCAGGATG-3′		
Bax-F	5′-TATGGGACACCAGGAGGGTA-3′	166	FJ977571.1
Bax-R	5′-CGTAGACCTTGCGGATAAAGC-3′		
ANXA2-F	5′-GGTGACTTCCGCAAGCTAATGG-3′	96	NM_205351.2
ANXA2-R	5′-CCTAGCGTCTTGGTCAATCAGTTC-3′		

### 2.9 Western-blot

At the designated time points, cells were lysed using RIPA buffer (Beyotime, China; Cat. P0013B) containing a protease and phosphatase inhibitor (Boster, China; Cat. AR1140), followed by continuous shaking on ice for 30 min. The supernatant was collected by centrifugation to obtain total protein lysates, and protein concentration was quantified using a BCA assay kit (Solarbio, China; Cat. PC0020). Protein samples were then separated on 12% or 15% SDS-PAGE gels (Boster, China; Cat. AR0138) and transferred to nitrocellulose membranes (Boster, China; Cat. AR0135-04). The membranes were blocked with 5% non-fat milk for 2 h. Subsequently, the membranes were incubated with specific antibodies, including B-cell lymphoma 2 (Bcl-2) (Proteintech, China; Cat. 12789-1-AP), Bcl-2-associated X protein (Bax) (Proteintech, China; Cat. 50599-2-1g), ANXA2 (Proteintech, China; Cat. 11256-1-AP), and β-actin (ABclonal, China; Cat. AC038) (loading control). Following this, the membranes were incubated with appropriate secondary antibody (ABclonal, China; Cat. AS014) for 45 min. Protein bands were visualized using an enhanced chemiluminescence reagent (Meilunbio, China; Cat. MA0186).

### 2.10 Determination of caspase-3 activity

Dynamic changes in caspase-3 activity were detected using a caspase-3 activity assay kit (Beyotime, China; Cat. C1115) according to the protocol. Cells were digested with 0.25% trypsin, centrifuged, and washed with PBS. Cell pellets were resuspended in lysis buffer (100 μL per 2 × 10^6^ cells) and incubated on ice for 15 min. After centrifugation at 12,000 × g for 15 min at 4°C, the supernatant was carefully collected. For enzymatic reactions, 50 μL of supernatant was mixed with 40 μL of lysis buffer and 10 μL of Ac-DEVD-pNA substrate (2 mM) in a 96-well plate. A blank control containing 50 μL of lysis buffer instead of sample was included. After incubation at 37°C for 2 h, absorbance at 405 nm (A405) was measured. The A405 value of pNA catalyzed by caspase-3 in the sample was calculated by subtracting the blank control absorbance from sample readings. Caspase-3 activity was quantified using a pNA standard curve (0~200 μM) and expressed as units per milligram of protein, where one unit corresponds to the hydrolysis of 1 nmol Ac-DEVD-pNA per hour at 37°C.

### 2.11 Statistical analysis

All data were presented as the mean ± standard deviation. Differences among groups were analyzed using one-way analysis of variance (ANOVA) and Tukey's multiple comparison test. A *P*-*value* of < 0.05 was considered significant. A *P*-*value* of < 0.01 was considered highly significant.

## 3 Results

### 3.1 Effects of *E. tenella* infection on ANXA2 expression

**Figure 1:**
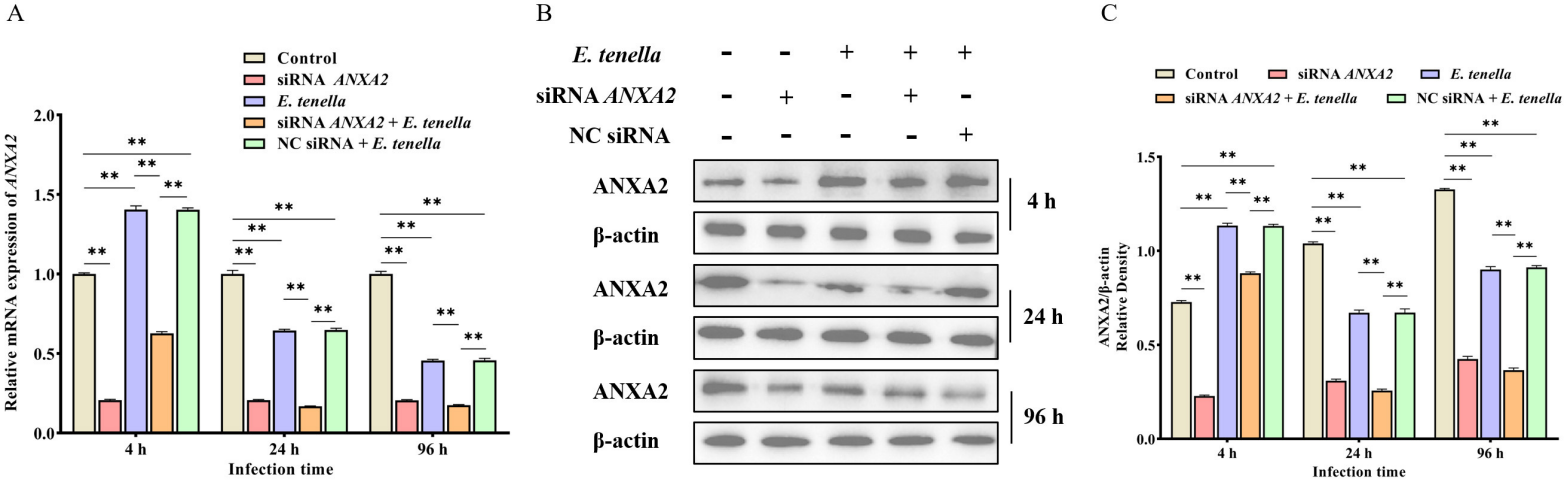
*E. tenella* infection affects the expression levels of ANXA2. **(A)** The relative expression levels of *ANXA2* mRNA in host cells infected with *E. tenella* sporozoites for 4 to 96 h. **(B,C)** The expression levels of ANXA2 proteins in host cells infected with *E. tenella* sporozoites for 4 to 96 h. **P < 0.01.

Western-blot and RT-qPCR analysis revealed that ANXA2 expression was significantly increased in both the *E. tenella* group and NC siRNA + *E. tenella* group at 4 h after inoculation but significantly decreased at 24 to 96 h when compared to the control group (Figure 1; *P* < 0.01). These result indicate that during the early phase of infection, *E. tenella* upregulates the expression of ANXA2, whereas it downregulates ANXA2 expression in the later stages of infection.

### 3.2 Knocking down the expression of ANXA2 reduces the infection rate of *E. tenella*

**Figure 2:**
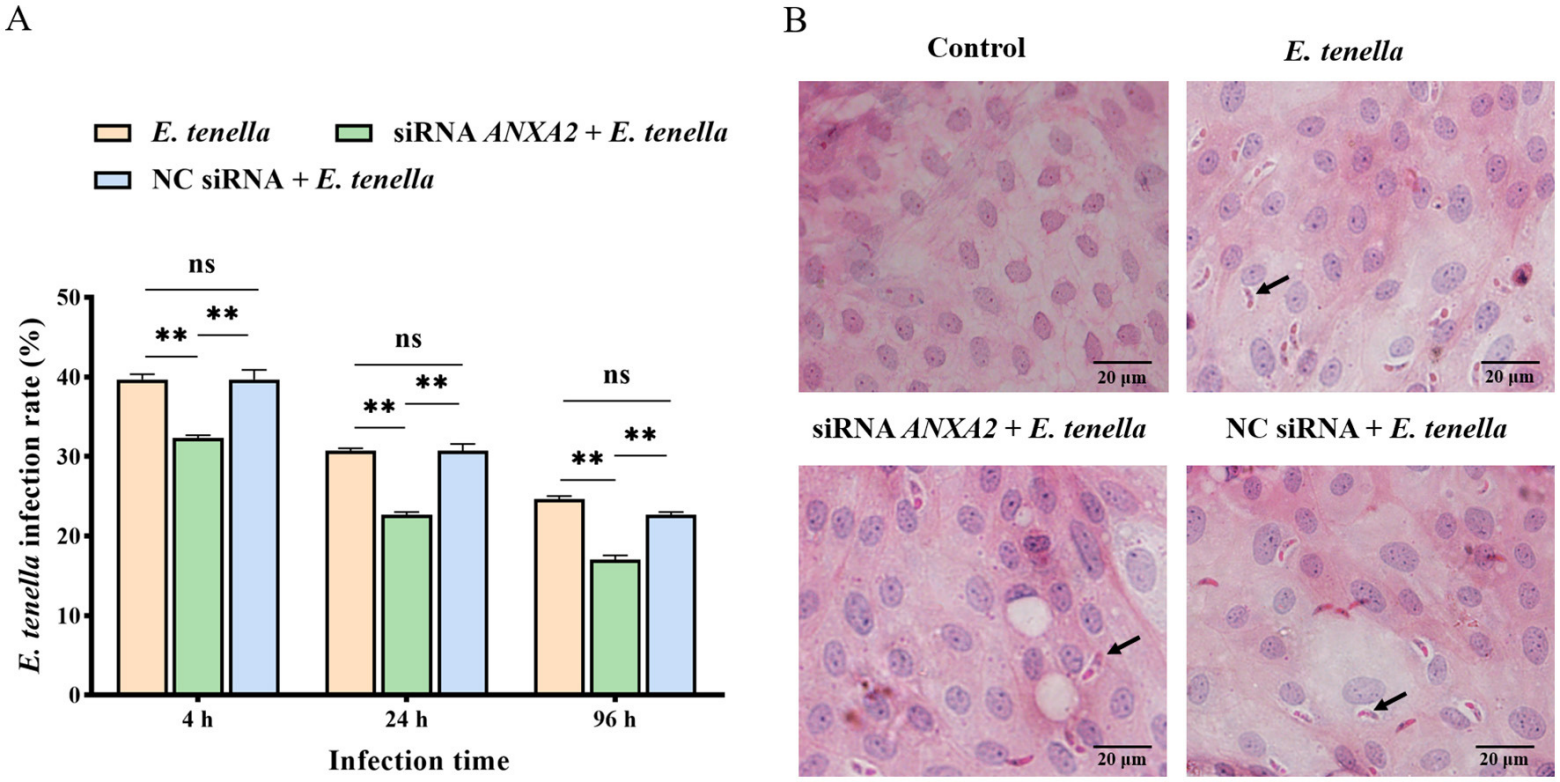
**(A)** Infection rate of cells at 4, 24, and 96 h after inoculation with *E. tenella*. **(B)** H.E staining of cells. The arrow points to the sporozoite. ns, *P* > 0.05, **P < 0.01.

Following sporozoite inoculation for 4 to 96 h, the infection rate in the siRNA *ANXA2* + *E. tenella* group was significantly lower than that in both the *E. tenella* and NC siRNA + *E. tenella* groups (*P* < 0.01; Figure 2). The result indicates that knocking down the ANXA2 expression can reduce the infection rate of *E. tenella*.

### 3.3 Knocking down the expression of ANXA2 increases the apoptosis rate in host cells of *E. tenella*

**Figure 3:**
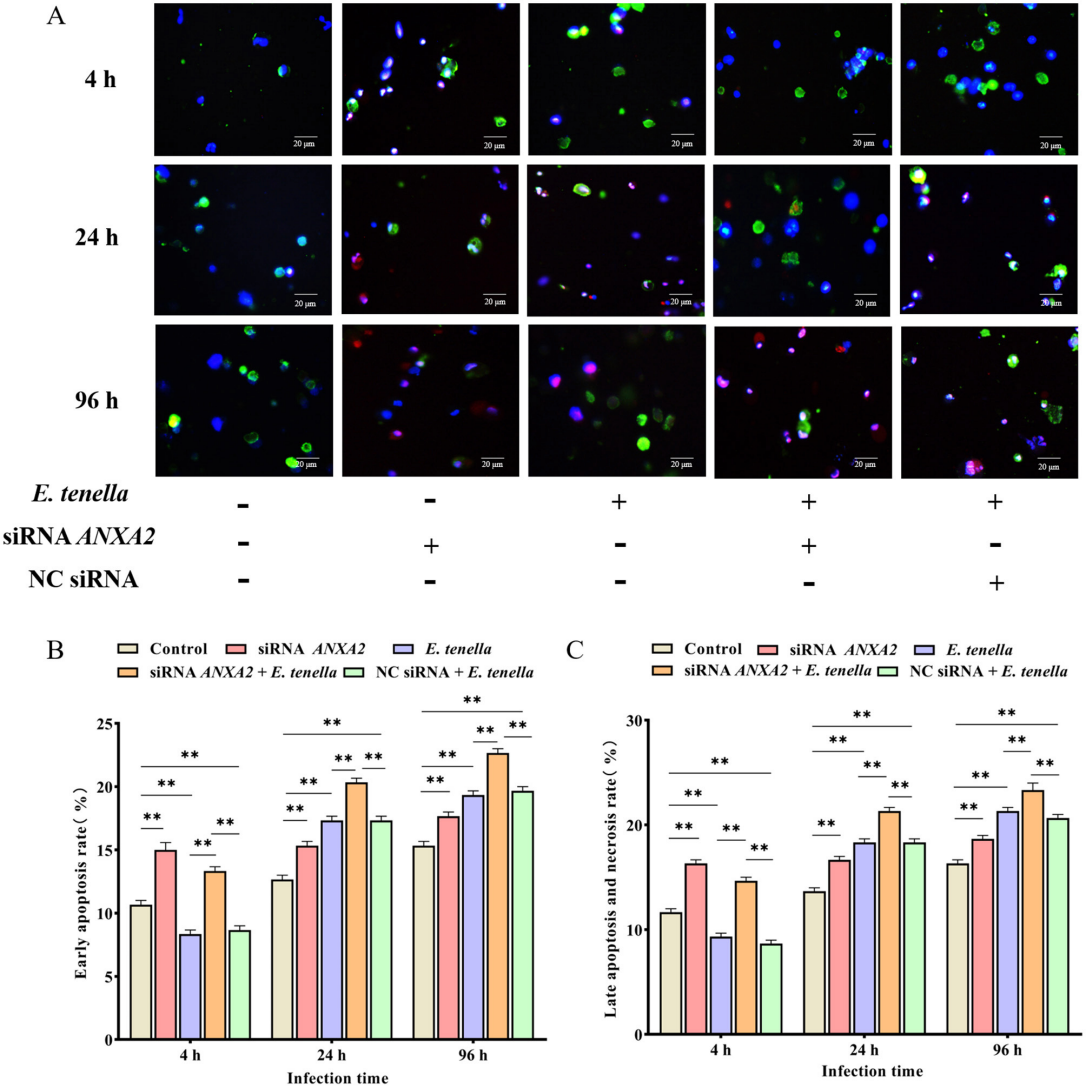
Apoptosis rate of chicken embryo cecal epithelial cells at 4, 24, and 96 h after inoculation with *E. tenella*. **(A)** Hoechst staining (blue) and Annexin V/PI staining (green/red) to detect apoptosis cells. Merge is Hoechst staining/Annexin V/PI staining overlay. **(B)** Early apoptosis rate of chicken embryo cecal epithelial cells inoculated with *E. tenella*. **(C)** Late apoptosis and necrosis rate of chicken embryo cecal epithelial cells inoculated with *E. tenella*. **P < 0.01.

At 4 h after sporozoite inoculation, compared to the control group, the rates of early apoptosis, late apoptosis, and necrosis of host cells in the *E. tenella* group were significantly reduced (*P* < 0.01), but significantly increased at 24 to 96 h (*P* < 0.01). After sporozoite inoculation for 4 to 96 h, the rates of early apoptosis, late apoptosis, and necrosis of host cells in the siRNA *ANXA2* group were significantly higher than those in the control group (*P* < 0.01). At 4 h after sporozoite inoculation, the rates of early apoptosis, late apoptosis, and necrosis of host cells in the siRNA *ANXA2* + *E. tenella* group were significantly lower than those in the siRNA *ANXA2* group (*P* < 0.05), but significantly higher than those in the *E. tenella* group and NC siRNA + *E. tenella* group (*P* < 0.01). At 24 to 96 h after sporozoite inoculation, the rates of early apoptosis, late apoptosis, and necrosis of host cells in the siRNA *ANXA2* + *E. tenella* group were significantly higher than those in the siRNA *ANXA2* group, *E. tenella* group and NC siRNA + *E. tenella* group (*P* < 0.01; Figure 3). These results indicate that ANXA2 knockdown significantly increases the apoptosis rates in host cells of *E. tenella*.

### 3.4 Knocking down the expression of ANXA2 increases the caspase-3 activity in host cells of *E. tenella*

**Figure 4:**
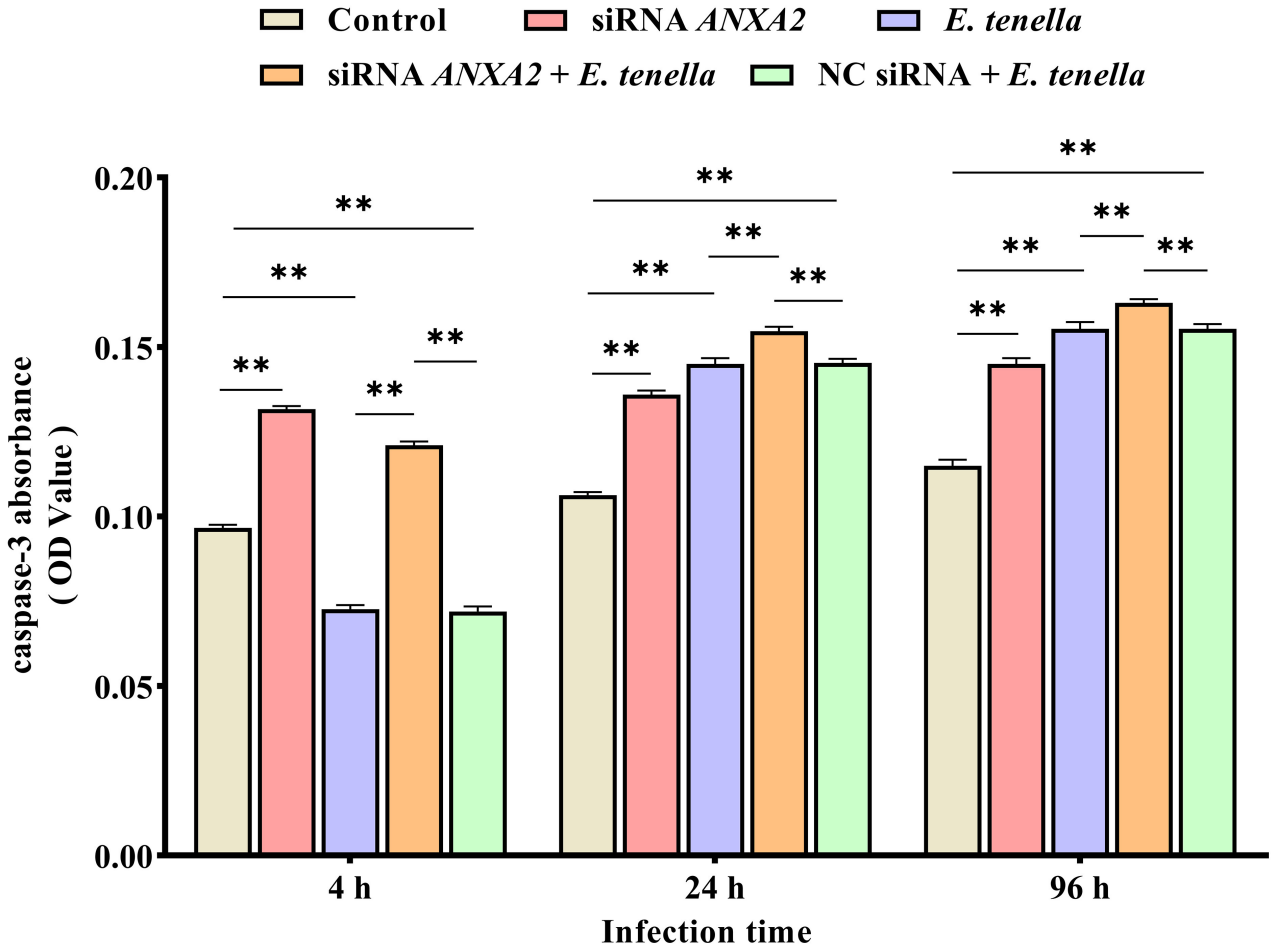
Caspase-3 activity of chicken embryo cecal epithelial cells inoculated with *E. tenella* for 4 to 96 h. **P < 0.01.

At 4 h after sporozoite inoculation, compared to the control group, the caspase-3 activity of host cells in the *E. tenella* group were significantly reduced (*P* < 0.01), but significantly increased at 24 to 96 h (*P* < 0.01). After sporozoite inoculation for 4 to 96 h, the caspase-3 activities of host cells in the siRNA ANXA2 group were significantly higher than that in the control group (*P* < 0.01). At 4 h after sporozoite inoculation, the caspase-3 activity of host cells in the siRNA ANXA2 + *E. tenella* group were significantly lower than that in the siRNA ANXA2 group (*P* < 0.01), but significantly higher than that in the *E. tenella* group and NC siRNA + *E. tenella* group (*P* < 0.01). At 24 to 96 h after sporozoite inoculation, the caspase-3 activity of host cells in the siRNA *ANXA2* + *E. tenella* group were significantly higher than that in the siRNA *ANXA2* group, *E. tenella* group and NC siRNA + *E. tenella* group (*P* < 0.01; Figure 4). These results indicate that ANXA2 knockdown significantly increases the caspase-3 activity in host cells of *E. tenella*.

### 3.5 Effects of ANXA2 knockdown on mRNA expression and protein activity of Bax and Bcl-2 in host cells of *E. tenella*

**Figure 5:**
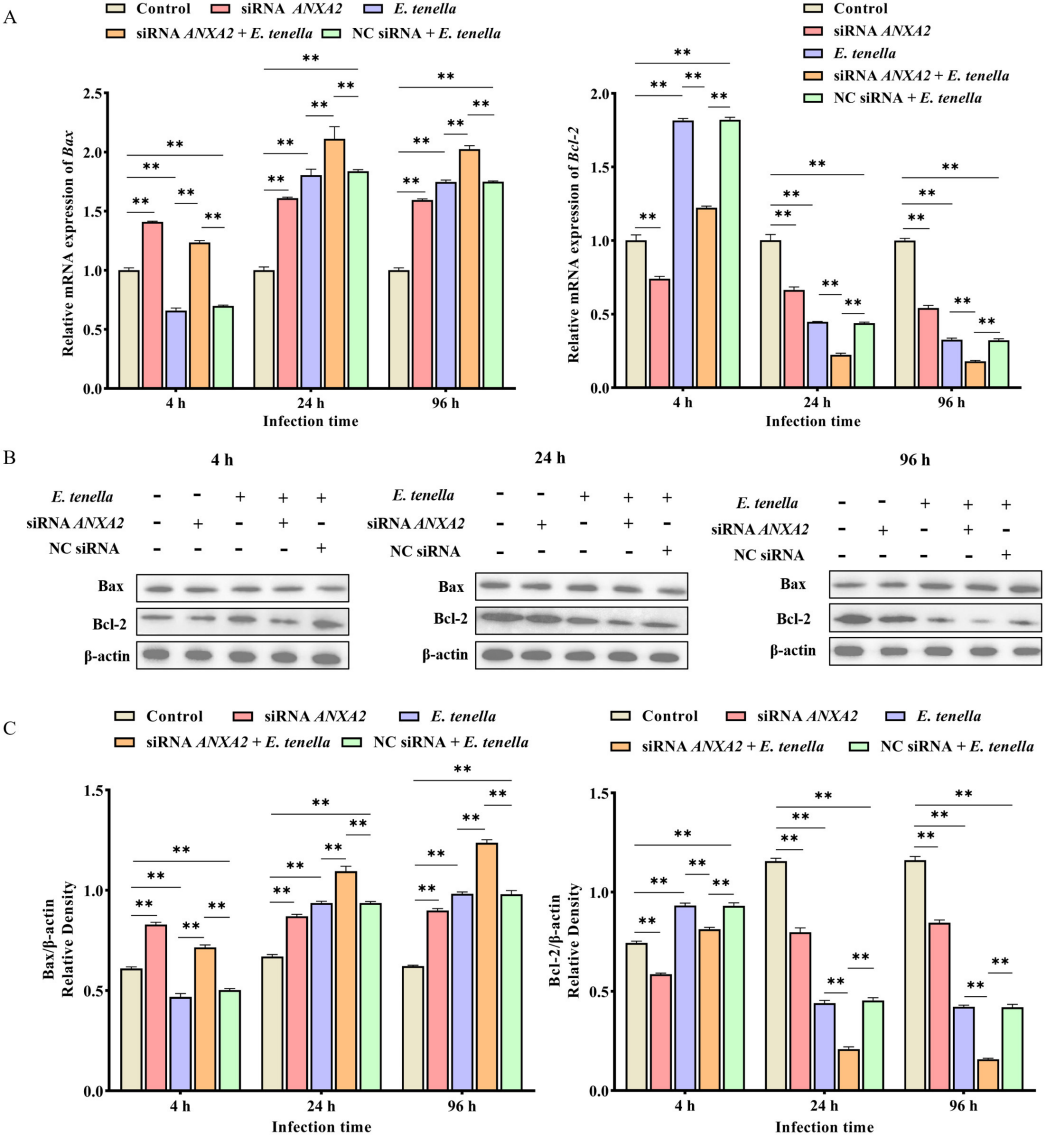
The expression changes of apoptosis-associated factors Bax and Bcl-2. **(A)** The relative expression levels of *Bax* and *Bcl-2* mRNA in host cells for 4 to 96 h. **(B,C)** The expression levels of Bax and Bcl-2 proteins in host cells for 4 to 96 h. **P < 0.01.

After sporozoite inoculation for 4 to 96 h, the expression level of *Bax* mRNA of host cells in the siRNA *ANXA2* group was significantly higher than that in the control group (*P* < 0.01), while the expression level of *Bcl-2* mRNA was significantly lower than that in the control group (*P* < 0.01); In the siRNA *ANXA2* + *E. tenella* group, the expression level of *Bax* mRNA of host cells was significantly higher than that in the *E. tenella* group and the NC siRNA + *E. tenella* group (*P* < 0.01). In contrast, the expression level of *Bcl-2* mRNA of host cells was significantly lower than that in the *E. tenella* group and the NC siRNA + *E. tenella* group (*P* < 0.01; Figure 5A). Western-blot results confirmed that the changes in protein activity of Bax and Bcl-2 consistent with the changes in their relative mRNA expression levels (Figures 5B, C). These results indicate that ANXA2 knockdown significantly upregulates the expression level of Bax and downregulates the expression level of Bcl-2 in host cells of *E. tenella*.

## 4 Discussion

ANXA2 is one of the most abundant proteins in biological cells and is also one of the most extensively studied members of the annexin superfamily (37–39). Previous reports have demonstrated that ANXA2 plays unique roles in the infection mechanisms of various pathogens (28). RNAi is a biological process initiated by double-stranded RNA (dsRNA), which induces sequence-specific degradation of complementary RNA through a process mediated by siRNA, ultimately resulting in post-transcriptional silencing of target gene expression (40). In this study, we successfully knocked down the expression of ANXA2 using RNAi and investigated its effects on apoptosis in host cells exposed to *E. tenella* sporozoites. Our results revealed that knocking down the expression of ANXA2 reduces the infection rate of *E. tenella*. ANXA2 may be involved in the invasion of chicken embryo cecal epithelial cells by *E. tenella*. Similarly, the protein ADP-ribosylation factor 6 (ARF6) facilitates the rearrangement of intracellular actin and endocytosis by binding to ANXA2 in *Trypanosoma cruzi* infection, assisting the parasite in invading host cells (41). Silencing the expression of ANXA2 markedly inhibits the replication of pseudorabies virus (PRV) in PK-15 cells (42). ANXA2 knockdown significantly reduces classical swine fever virus (CSFV) production (43). The biphasic expression pattern of ANXA2, characterized by an initial increase followed by a subsequent decline, is intriguing and may be associated with the stages of parasite development or host cell signaling responses. During the early stages of infection, the upregulation of ANXA2 could facilitate parasite invasion by interacting with specific parasite proteins, as seen in other host-pathogen interactions. As the infection progresses, the subsequent downregulation of ANXA2 might reflect a host response aimed at limiting parasite replication or a shift in the host cell's signaling pathways to promote apoptosis and limit further spread of the infection.

During the early stage of infection, *E. tenella* suppresses host cell apoptosis, whereas in the mid-to-late stages, it promotes apoptotic processes (44). Our findings indicated that the apoptosis rate decrease at 4 h post-infection with *E. tenella*, but significantly increase at 24 h and 96 h, which is consistent with the previous study. In addition, ANXA2 silencing enhances the caspase-3 activity in host cells. Caspase-3, as a key executor of apoptosis, marks the beginning of programmed cell death upon its activation (45). The correlation between ANXA2 expression and caspase-3 activity emphasizes the importance of ANXA2 in the regulation of the apoptotic pathways. Another important discovery of this study is the differential regulation of apoptotic genes in host cells by ANXA2. Bax and Bcl-2 are key regulatory factors of the intrinsic apoptosis pathway, modulating cell apoptosis by controlling mitochondrial permeability (46, 47). Suppressing ANXA2 leads to a significant decrease in the expression of the anti-apoptotic protein Bcl-2, while simultaneously increasing the expression of the pro-apoptotic protein Bax. These findings showed that ANXA2 participates in the apoptosis process induced by *E. tenella*, and knockdown of ANXA2 promotes cell apoptosis, which is in agreement with previously reported functions of ANXA2 in apoptosis. For example, ANXA2 knockdown leads to an increase in radiation-induced apoptosis in radioresistant nasopharyngeal carcinoma (NPC) cells, whereas an elevation in ANXA2 expression leads to a decrease in radiation-induced apoptosis in NPC cells (48); the 2A protein from the encephalomyocarditis virus inhibits the JNK/c-Jun signaling pathway through its interaction with ANXA2, facilitating apoptosis in BHK21 cells during the early phases of virus replication (49). While the increase in apoptosis via the Bax/Bcl-2 and caspase-3 pathways following ANXA2 knockdown is well documented in our study, the mechanistic connection between ANXA2 and these apoptotic markers remains largely correlative. ANXA2 is known to be involved in various cellular processes, including membrane repair, calcium signaling, and immune modulation (50). It is plausible that ANXA2's role in apoptosis during *E. tenella* infection involves its interactions with other cellular proteins and signaling pathways. For example, ANXA2 has been shown to activate the JNK pathway, which is involved in apoptosis (34). Additionally, ANXA2's involvement in calcium signaling could influence mitochondrial permeability and subsequent activation of the intrinsic apoptosis pathway (26).

This study elucidates the role of ANXA2 in modulating apoptosis during *E. tenella* infection, providing novel insights into host-parasite interactions. While our findings highlight ANXA2 as a potential therapeutic target, several limitations warrant consideration. For instance, the reliance on *in vitro* models may not fully recapitulate the complexity of *E. tenella* infection *in vivo*, where host immune responses and microenvironmental factors could alter ANXA2-mediated pathways (26, 51). Furthermore, the pleiotropic functions of ANXA2 in cellular processes raise concerns about off-target effects if ANXA2 inhibition is pursued as a therapeutic strategy (52, 53). Therefore, future studies should define how ANXA2 regulates apoptosis and test the efficacy and safety of ANXA2-targeted treatments *in vivo* to develop new strategies against *E. tenella* infection.

## 5 Conclusion

In conclusion, the infection caused by *E. tenella* influences ANXA2 expression. Knocking down ANXA2 expression significantly decreases *E. tenella* infection rates and increases host cell apoptosis. The findings of this study establish a basis for additional research into the pathogenic mechanisms of *E. tenella* evasion and host pathogenesis, showing that targeting ANXA2 may be a viable approach for controlling coccidiosis.

## Data Availability

The original contributions presented in the study are included in the article/Supplementary material, further inquiries can be directed to the corresponding author.
