# Supplementary material for: Knockdown of annexin A2 enhances the host cell apoptosis induced by Eimeria tenella
Source: Front Vet Sci. 2025 Jul 24;12:1595384. doi: 10.3389/fvets.2025.1595384 (PMC12330289; doi:10.3389/fvets.2025.1595384)
Supplement: Supplementary file 1 [file Data_Sheet_1.zip › Supplementary material presentation/List of Abbreviations.docx]

List of Abbreviations

| Abbreviation | Full Form |
| --- | --- |
| **ANOVA** | Analyzed using one-way analysis of variance |
| **ANXA2** | Annexin A2 |
| **ARF6** | ADP-ribosylation factor 6 |
| AIV | Avian influenza virus |
| **Bax** | Bcl-2-associated X protein |
| **Bcl-2** | B-cell lymphoma 2 |
| **CSFV** | Classical swine fever virus |
| **dsRNA** | Double-stranded RNA |
| ***E. tenella*** | *Eimeria tenella* |
| **EGF** | Epidermal Growth Factor |
| **EGFR** | Epidermal growth factor receptor |
| ***Et*Serpin1** | *Eimeria tenella* serine protease inhibitor 1 |
| **FBS** | Fetal bovine serum |
| **H&E** | Hematoxylin and eosin |
| **JNK** | c-Jun N-terminal kinase |
| **MAPK** | Mitogen-activated protein kinase |
| **NC** | Negative control |
| **NPC** | Nasopharyngeal carcinoma |
| **PRRSV** | Porcine reproductive and respiratory syndrome virus |
| **PRV** | Pseudorabies virus |
| **RNAi** | RNA interference |
| **siRNA** | Small interfering RNA |
| **SPF** | Specific-pathogen-free |
