# Supplementary material for: Knockdown of annexin A2 enhances the host cell apoptosis induced by Eimeria tenella
Source: Front Vet Sci. 2025 Jul 24;12:1595384. doi: 10.3389/fvets.2025.1595384 (PMC12330289; doi:10.3389/fvets.2025.1595384)
Supplement: Supplementary file 1 [file Data_Sheet_1.zip › Supplementary material presentation/Table 1.docx]

**Table 1.** siRNA sequence**.**

| Name |  | Sequence |
| --- | --- | --- |
| siRNA ANXA2 | Sense | GAUGCUGGUGUCAAGAGAATT |
|  | Antisense | UUCUCUUGACACCAGCAUCTT |
| NC siRNA | Sense | UUCUCCGAACGUGUCACGUTT |
|  | Antisense | ACGUGACACGUUCGGAGAATT |
