# Supplementary material for: Knockdown of annexin A2 enhances the host cell apoptosis induced by Eimeria tenella
Source: Front Vet Sci. 2025 Jul 24;12:1595384. doi: 10.3389/fvets.2025.1595384 (PMC12330289; doi:10.3389/fvets.2025.1595384)
Supplement: Supplementary file 1 [file Data_Sheet_1.zip › Supplementary material presentation/Table 2.docx]

**Table 2.** Sequences of the primers used for the qRT-PCR assay.

| Gene name | Primer sequences | Product length(bp) | GenBank  accession No. |
| --- | --- | --- | --- |
| β-actin-F | 5’-CACCACAGCCGAGAGAGAAAT-3’ | 135 | L08165.1 |
| β-actin-R | 5’-TGACCATCAGGGAGTTCATAGC-3’ |  |  |
| Bcl-2-F | 5’-AGGACAACGGAGGATGGGATG-3’ | 109 | NM_205339 |
| Bcl-2-R | 5’-ACCAGAACCAGGCTCAGGATG-3’ |  |  |
| Bax-F | 5’-TATGGGACACCAGGAGGGTA-3’ | 166 | FJ977571.1 |
| Bax-R | 5’-CGTAGACCTTGCGGATAAAGC-3’ |  |  |
| Annexin A2-F | 5’-GGTGACTTCCGCAAGCTAATGG-3’ | 96 | NM_205351.2 |
| Annexin A2-R | 5’-CCTAGCGTCTTGGTCAATCAGTTC-3’ |  |  |
